# COVID-19 Infection among Older People Admitted to Hospital: A Cross-Sectional Analysis

**DOI:** 10.3390/geriatrics6010025

**Published:** 2021-03-08

**Authors:** Chiann Ni Thiam, Kejal Hasmukharay, Wan Chieh Lim, Chai Chen Ng, Gordon Hwa Mang Pang, Aimy Abdullah, Nor Izzati Saedon, Hui Min Khor, Terence Ong

**Affiliations:** 1 Department of General Medicine, Hospital Kuala Lumpur, Ministry of Health, Jalan Pahang, Kuala Lumpur 50586, Malaysia; cnthiam617@gmail.com (C.N.T.); limwanchieh@gmail.com (W.C.L.); chaichen257@hotmail.com (C.C.N.); gordonpang84@yahoo.com (G.H.M.P.); 2 Geriatric Medicine Unit, Department of Medicine, University Malaya Medical Centre, Kuala Lumpur 59100, Malaysia; kejal_has@yahoo.com (K.H.); aimyabdullah@gmail.com (A.A.); izzati@ummc.edu.my (N.I.S.); huimin81@yahoo.com (H.M.K.); 3 Faculty of Medicine, University of Malaya, Kuala Lumpur 50603, Malaysia; 4 Faculty of Medicine, Universiti Teknologi MARA, Sungai Buloh Campus, Jalan Hospital, Sungai Buloh 47000, Malaysia

**Keywords:** aged, COVID-19, hospital, Malaysia

## Abstract

(1) Background: Older people with COVID-19 infection report worse clinical outcomes. There is a paucity of local data and this study aimed to describe the clinical progression of older people admitted to a university hospital in Malaysia with COVID-19 infection. (2) Methods: Older people (≥60 years) admitted with COVID-19 infection confirmed with RT-PCR from 27 February 2020–25 May 2020 were included in this study. Data on patient characteristics, hospital treatment, and inpatient outcomes were collected via hospital-held electronic medical records. Analysis was done to describe the cohort and identify factors associated with inpatient mortality. (3) Results: 26 participants were included (mean age 76.2 years, female 57.7%). All had at least one comorbid condition and half were frail. About 19.2% had non-respiratory (atypical) symptoms; 23.1% had a severe disease that required intensive care unit monitoring; 46.2% were given COVID-19 targeted therapy. Inpatient mortality and overall complication rates were 23.1% and 42.3%, respectively. Delirium on presentation and lower Ct-value were associated with mortality. (4) Conclusions: Older people with COVID-19 infection have severe infection and poor hospital outcomes. Vigilant hospital care is necessary to address their multimorbidity and frailty, along with appropriate treatment for their infection.

## 1. Introduction

Worse clinical outcomes were observed among those aged 65 years and above with COVID-19 infection. The case fatality rate was reported to be as high as 14.8–20.2% in those aged 80 years and above [1,2]. In Malaysia, there are currently 100,318 reported cases, with 446 deaths, for a total case fatality rate of 0.45% [3,4]. However, 64% of these reported deaths involved an older person [3,4]. Older people were at least 2.5 times more likely to require intensive care or die, as compared to their younger counterparts [5]. Many have diminished physiological reserve, multimorbidity, frailty, and disability which likely contributes to these poor outcomes. Hospital treatment, prolonged bed rest, and isolation precautions might increase the risk of other adverse outcomes, such as deconditioning, delirium, hospital-acquired infection, and pressure sore.

Furthermore, atypical presentation of COVID-19 infection such as delirium is commonly described among older people [6,7]. Older persons were less likely to present with respiratory symptoms [6,7]. This poses a diagnostic challenge that leads to potential treatment delay. Several deaths due to COVID-19 infection outside the hospital were reported in Malaysia and usually involved an older person [4]. Hence, COVID-19 also highlighted long-standing barriers to healthcare [8].

Despite COVID-19 showing the greatest impact on an older person, limited data exist on their specific demographics, treatment, and outcomes in Malaysia. It is important to recognize other hospital treatment for older persons and their healthcare outcomes, as the national response to the pandemic needs to be driven by data specific to its context. This study aimed to describe the characteristics, presentation, treatment, and hospital-specific healthcare outcomes of the older person admitted for COVID-19 infection to a university hospital in Malaysia.

## 2. Materials and Methods

This is a retrospective study of Malaysian nationality aged 60 years old and above, diagnosed with COVID-19 infection, and treated in University Malaya Medical Centre (UMMC) from 25 February 2020 till 27 May 2020. UMMC is a university hospital serving a local catchment area of 300,000 people and is one of the 60 designated COVID-19 hospitals in Malaysia.

Data on routine clinical information of hospital admission was collected via electronic medical records (EMR). Data collected on patient’s characteristics included their demographics, residential status, ability to perform all basic activities of daily living (ambulating, feeding, dressing, personal hygiene, continence, toileting), frailty described according to the Clinical Frailty Scale (Clinical Frailty Scale ≥ 4 was identified as frail) [9], and comorbidities.

**Figure 1:**
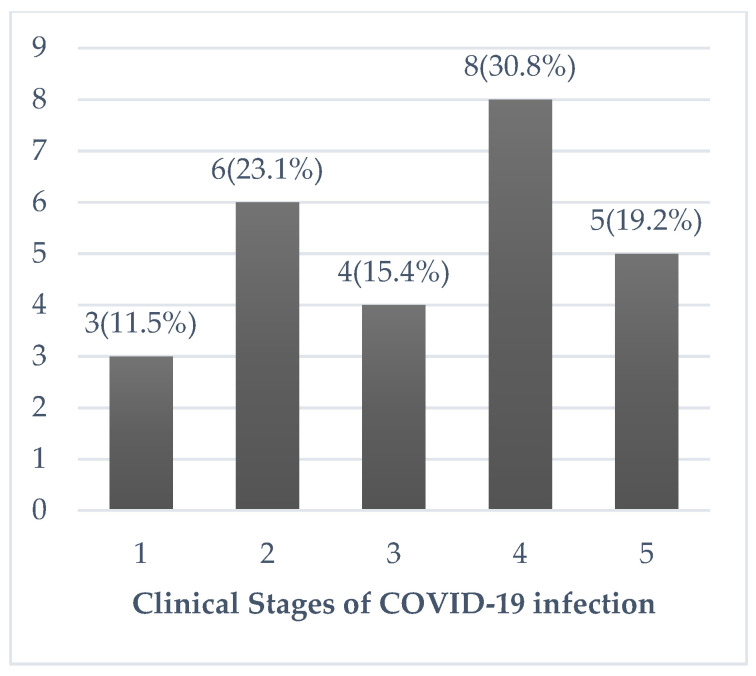
Clinical Stages of COVID-19 infection classification according to the Ministry of Health Malaysia. Stage 1: Asymptomatic. Stage 2: Symptomatic without radiological pneumonia changes. Stage 3: Symptomatic with radiological pneumonia changes. Stage 4 Symptomatic, pneumonia and supplemental oxygen required. Stage 5: Critically ill with multi-organ failure.

To confirm the diagnosis of COVID-19 infection, oropharyngeal and nasopharyngeal swabs were obtained and tested by real-time polymerase chain reaction (RT-PCR) assay, which is indicated by the cycle threshold (Ct). A positive test was defined as a Ct-value <40. Ct-value is inversely proportional to SARS-CoV-2 viral load. SARS-CoV-2 is the virus implicated in COVID-19 infection. Information on COVID-19 presentation (atypical presentation refers to being symptomatic but without cough, fever, or dyspnea [6]), the clinical severity according to classification by the Ministry of Health (Figure 1), radiological findings and laboratory parameters were recorded, based on what was documented in EMR. The Ministry of Health classifies the disease as follows. Stage 1—asymptomatic; Stage 2—symptomatic without radiological pneumonia changes; Stage 3—symptomatic with radiological pneumonia changes; Stage 4—symptomatic, pneumonia, and supplemental oxygen required; and Stage 5—critically ill with multi-organ failure [4]. The use of COVID-19-targeted therapy, including hydroxychloroquine, lopinavir/ritonavir, tocilizumab, interferon, and steroid, as well as concurrent administration of antibiotics were reported. The adverse outcomes studied were in-patient mortality and complications, such as hospital-acquired pneumonia, venous thromboembolism (VTE), pressure sore, and delirium.

Descriptive statistics were reported as mean with standard deviation (SD) for normally distributed data and median with interquartile range (Q1–Q3) for non-normally distributed data. Categorical data were presented as numbers with percentages. Chi-squared tests were used to study the association between categorical variables. The mean differences between two sets of numerical variables were assessed using appropriate statistics based on their distribution. Patient characteristics, presenting symptoms, laboratory parameters, and treatment administered were analyzed for their association with inpatient mortality, with appropriate statistical tests. Analyses were only performed on available data. Statistical significance was defined as a *p*-value of less than 0.05. Analyses were performed using IBM SPSS Statistics v.26.0. Ethical approval was obtained from the University of Malaya Research Ethics Committee (MREC ID NO: 2020428-8574).

## 3. Results

A total of 26 patients were diagnosed with COVID-19 infection in UMMC, during the 3-month study period, with a mean age of 76.2 (8.2) years. Majority were women (15/26 patients, 57.7%) and all patients had at least one concurrent comorbid diagnosis. Seventeen (65.4%) patients acquired Covid-19 infection from the community, whereas nine (34.6%) patients contracted the infection whilst hospitalized. Patient baseline characteristics are shown in Table 1.

The median of symptom onset to admission was 5 (IQR 1–10) days. Twenty-three patients (85.5%) were symptomatic and five (19.2%) patients had atypical presentations. No patient reported anosmia. Regarding chest radiograph findings on admission, 13 (50.0%) patients had infiltrates typical of COVID-19 infection, five (19.2%) had pleural effusion or interstitial changes not typical of COVID-19 infection, six (23.1%) had normal chest radiograph, and data was not recorded in two (7.7%) patients.

The clinical stages of the patients were demonstrated in Figure 1. Six (23.1%) patients with Stage 4/5 illness were monitored in ICU. Three (11.5%) and two (7.7%) patients required invasive and non-invasive ventilation, respectively. Four (15.4%) patients required inotropic support. Five (19.2%) patients developed Acute Respiratory Distress Syndrome (ARDS), and one (3.8%) developed myocarditis. Twelve (46.2%) patients—Stage 5: 5/5 (100%) patients; Stage 4: 3/8 (37.5%) patients; Stage 3: 3/4 (75.0%) patients; Stage 2: 1/5 (20.0%) patients, were given COVID-19-targeted therapy. Among these, ten (38.5%) patients received 400 mg of hydroxychloroquine twice a day for one day, followed by 200 mg twice a day for a mean duration of 4.9 (2.3) days; six (23.1%) received two tablets of 200 mg/50 mg of lopinavir/ritonavir twice a day for a median duration of 2.5 (2.0–4.8) days; four (15.4%) received a single dose of 8 mg/kg of tocilizumab; one (3.8%) received 2 doses of Interferon beta-1b 250 mcg; and two (7.7%) received steroids, which were dexamethasone or hydrocortisone. Eighteen (69.2%) patients received concurrent antibiotics. All 13 (50.0%) patients with a Clinical Frailty Scale ≥4 had a “Do Not Attempt Resuscitation” order in place, during their hospitalization. Twelve of these frail patients did not receive COVID-19-targeted therapy; of which, seven were asymptomatic or had mild disease, and medical treatment was deemed futile in five patients. Nine (34.6%) patients received pharmacological venous thromboembolism prophylaxis.

The median (IQR) length of stay was 27.5 (15.0–40.5) days. Six (23.1%) patients died in hospital and five received specialist palliative care input. Eleven (42.3%) patients developed inpatient complications. Delirium was the commonest inpatient complication. Ten (38.5%) patients developed delirium during their hospitalization, four (15.4%) patients developed hospital-acquired pneumonia, four (15.4%) patients developed pressure sores, and one (3.8%) patient developed a venous thromboembolic event.

**Figure 2:**
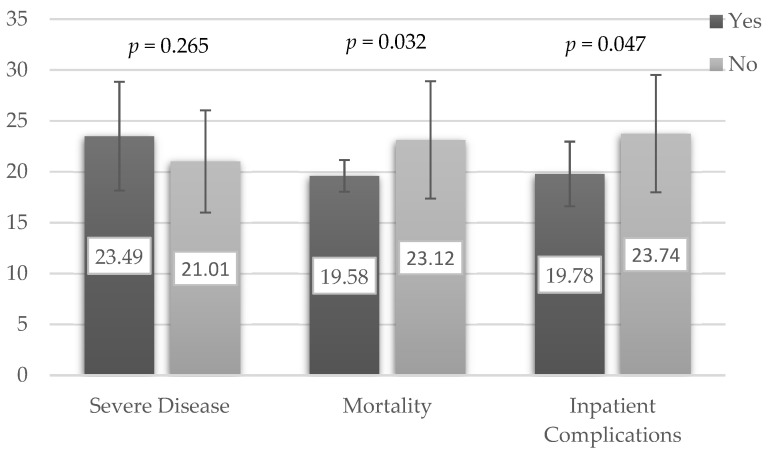
Mean Ct-Value (Standard Deviation) according to disease severity and adverse clinical outcomes. Severe Disease—clinical stage 4 and 5. Inpatient complications—mortality, delirium, hospital-acquired pneumonia, pressure sore, or venous thromboembolism.

Delirium was the only presenting symptom that was associated with mortality (Table 1). There was no association between mortality and patient demographics, comorbidities, and treatment received (Table 1). Apart from the low Ct-value, other laboratory parameters were not associated with mortality (Table 1). The lowest Ct-value recorded on average was 7 (6) days, after the onset of symptoms. Ct-value was also significantly lower in patients who died and those who developed inpatient complications (Figure 2). However, this was not shown in patients with severe disease (Figure 2).

## 4. Discussion

This is the first study to specifically report on the characteristics and outcomes of older people admitted to hospital with COVID-19 infection in Malaysia. All patients in this cohort were living with at least one comorbid condition. Many were frail, required assistance with personal activities of daily living and help with their mobility. Cough was the commonest presenting symptoms but nonspecific symptoms, such as lethargy, loss of appetite, and delirium, were also common. Apart from Ct-value, none of the studied laboratory parameters were associated with mortality. In this cohort, the mortality rate and adverse outcome rates were 23.1% and 42.3%, respectively.

The severity of the infection and age-specific mortality rate reported in this study were similar to what was reported [10,11,12,13]. Delirium is recognized to be a key presenting feature for COVID-19 [14]. In this study, the frequency of delirium on presentation and its association with mortality was in keeping with other hospital-based studies [11,14,15,16]. Failure to recognize delirium as an important symptom of COVID-19 infection, by both family members and clinicians, might contribute to late presentation and delayed treatment, which might explain worse outcomes among older patients with delirium [15]. Although virus-mediated inflammatory response was the suggested mechanism of COVID-19-associated encephalopathy [17], delirium in older persons tends to be multifactorial [18]. Delirium is a result of complex inter-relationship between the patient’s baseline vulnerability (comorbidity, frailty, and disability), and exposure to noxious insults (COVID-19 infection and its associated complications) [18].

Ct-value, which was used as a surrogate marker of viral load, was weakly associated with mortality and inpatient complications, but not disease severity. This finding was consistent with the existing literature [19,20,21,22]. Nevertheless, analysis of the Ct-value needs to be interpreted with caution. In this study, the lowest recorded Ct-value was analyzed instead of the baseline Ct-value, as in other studies that were generally taken on the day of admission [21,22]. The onset of illness to admission ranged from 1 to 10 days. As repeating RT-PCR was not routinely performed in this hospital, the lowest Ct-value might not accurately indicate the peak viral load, as the actual lowest Ct-value might not be captured. Additionally, this study was unable to evaluate how the change in Ct-value would affect the disease severity and its clinical course. The timing from the onset of symptoms to RT-PCR test was recommended to be standardized in evaluating the association of Ct values with disease severity, as viral load peaked before day 5 in COVID-19 infection [23,24].

The findings of this study were limited by the small patient numbers and its dependency on the data entered into the hospital’s EMR system. The diagnosis of delirium, which was associated with mortality in this study, was dependent on clinical entry, and information was lacking if any accepted diagnostic criteria were used. Another key data that was not part of this data set was body mass index. There is emerging scientific literature that patients who were obese were more susceptible to COVID-19 infection and would be vulnerable to poor outcomes [25]. This appears multifactorial, due to a combination of metabolic dysfunction, impaired immune response, and adipose-related inflammation [25]. At the time of the study, the number of COVID-19 cases was low and the majority were treated at another hospital that was designated as the main COVID-19 care center in Malaysia. This study did include all older patients who were admitted during the study period, which meant that there was no selection bias. This study was from a single site in an urban area. Hence, the patient characteristics and care delivered might not be generalizable to other hospital settings. However, the management delivered by this hospital adhered to a wider treatment consensus agreed with other COVID-19 hospitals and guidelines produced by the Ministry of Health. Therefore, its practice should not be different from elsewhere.

Older patients [26,27], living with dementia [28] or with multimorbid conditions, tend to have poor outcomes associated with COVID-19 infection. This cohort was more likely to reside in institutionalized care. Care homes can be a nidus for COVID-19 infection and local outbreaks were reported [29]. Treating older people with COVID-19 infection in hospital is challenging, due to both intrinsic factors (multiple comorbidities, frailty, disability, and atypical presentation) and extrinsic factors (health care accessibility, resources limitation, and ageism). This led to an ethical discussion on how we can ensure that the pandemic is best treated, in light of resource limitation [30]. To support better care for older people, a frailty-based triage framework showed promising results [31]. When palliation is chosen, technology can be used to stay connected with the loved one, despite isolation precaution, although it is not perfect [32].

As Malaysia currently deals with its more serious third wave, the number of older people admitted to hospital with COVID-19 would increase. Based on the description of this study’s cohort and the treatment provided, older people with COVID-19 in hospital requires input from different specialist services, from geriatric medicine, critical care, palliative care, and rehabilitation services. Their care needs to be different, as they are more likely to present without typical respiratory symptoms, such as more likely to present with delirium, and have worse outcomes compared to a younger cohort. The challenge of treating someone with COVID-19 infection, which includes physical distancing from friends and family, protective equipment, and communication barrier, adds an extra toll to these patients’ care. Humanizing the care delivered throughout the hospital journey and bearing in mind the overall impact COVID-19 has had on the patient and their carers is just as important as the medical intervention provided [33]. Just as how treatment for COVID-19 evolved since the start of the pandemic, how care for older people with COVID-19 is organized and coordinated also needs to evolve. The findings of this study will provide the platform for this change.

## Figures and Tables

**Table 1:** Patient baseline characteristics.

	All, N = 26	Alive, N = 20	Death, N = 6	
	*n* (%)	*n* (%)	*n* (%)	*p*
Age, mean (SD) years	76.2(8.2)	74.5(7.6)	81.8(8.0)	0.053
Female	15(57.7%)	12(60.0%)	3(50.0%)	>0.999
Nursing home resident	8(30.8%)	5(25.0%)	3(50.0%)	0.330
BADL ^a^ independent	15(57.7%)	13(65.0%)	2(33.3%)	0.348
Ambulation with aids/wheelchair	12(46.2%)	8(40.0%)	4(66.7%)	0.365
Clinical Frailty Scale ≥ 4	13(50.0%)	8(40.0%)	5(83.3%)	0.160
Hospital-acquired COVID-19	9(34.6%)	5(25.0%)	4(66.7%)	0.138
Comorbidities				
Diabetes Mellitus	16(61.5%)	14(70.0%)	2(33.3%)	0.163
Hypertension	22(84.6%)	19(95.0%)	3(50.0%)	0.028
IHD/CCF ^b^	5(19.2%)	5(25.0%)	0	0.298
Chronic kidney disease	5(19.2%)	3(15.0%)	2(33.3%)	0.558
Dementia	8(30.8%)	4(20.0%)	4(66.7%)	0.051
Stroke	8(30.8%)	5(25.0%)	3(50.0%)	0.330
Charlson Comorbidity Index, median (IQR)	6.0(3.5–7.0)	6.0(3.0–7.0)	5.5(4.0–7.3)	0.458
Use of ARB/ACE-I ^c-^ before admission	12(46.2%)	11(55.0%)	1(16.7%)	0.170
Presenting symptoms				
Symptomatic on admission	23(88.5%)	17(85.0%)	6(100.0%)	>0.999
Fever	11(42.3%)	8(40.0%)	3(50.0%)	>0.999
Respiratory symptoms ^d^	14(53.8%)	12(60.0%)	2(33.3%)	0.365
Gastrointestinal symptoms ^e^	9(34.6%)	9(45.0%)	0	0.063
Loss of appetite	10(38.5%)	9(45.0%)	1(16.7%)	0.352
Lethargy	10(38.5%)	6(30.0%)	4(66.7%)	0.163
Delirium on presentation	7(26.9%)	3(15.0%)	4(66.7%)	0.028
Laboratory parameters				
Haemoglobin, g/dL, mean (SD)	12.15(2.48)	12.09(1.91)	12.38(4.27)	0.889
White cell count ×10^9^ L, median (Q1–Q3)	6.90(5.90–9.90)	6.60(5.30–7.88)	8.10(6.16–13.10)	0.628
Absolute lymphocyte count ×10^9^ L^,^ median (Q1–Q3)	1.28(1.03–2.11)	1.41(1.11–2.29)	1.00(0.91–1.41)	0.061
Absolute neutrophil count ×10^9^ L, median (Q1–Q3)	4.02(3.11–6.60)	3.93(2.68–5.21)	6.60(4.62–10.58)	0.031
Urea, mmol/L, median (Q1–Q3)	6.8(5.3–12.4)	6.9(4.4–15.3)	8.0(6.3–13.0)	0.724
Creatinine, mmol/L median (IQR)	78.0(68.0–103.0)	89.0(52.5–114.5)	78.0(73.5–149.5)	0.667
Albumin, g/L, mean (SD)	29.0(7.6)	29.6(5.9)	26.8(12.0)	0.079
Ferritin, ng/mL, median (Q1–Q3)	561(360–1174)	556(360–784)	1176(574–2224)	0.090
C-Reactive Protein, mg/L, median (Q1–Q3)	21.3(11.4–127.4)	19.9(11.4–39.2)	168.0(39.1–211.7)	0.073
Lowest Ct-value ^f^, mean(SD)	22.35(5.29)	23.11(5.77)	19.58(1.56)	0.032
Treatment				
Concomitant antibiotic use	18(69.2%)	13(65.0%)	5(83.3%)	0.628
COVID-19 targeted therapy	12(46.2%)	11(55.0%)	1(16.7%)	0.170
VTE ^g^ prophylaxis	9(34.6%)	8(40.0%)	1(16.7%)	0.380

^a^ Basic Activities of Daily Living. ^b^ Ischemic heart disease/congestive cardiac failure. ^c^ Angiotensin receptor blocker/angiotensin-converting enzyme inhibitor. ^d^ Cough, sore throat, and shortness of breath. ^e^ Nausea, vomiting, diarrhea, and abdominal pain. ^f^ Cycle threshold. ^g^ Venous thromboembolism.

## Data Availability

Data available only on request, due to privacy concerns and ethical restrictions.
